# Evaluating Impact on Motivation and Academic Performance of a Game-Based Learning Experience Using Kahoot

**DOI:** 10.3389/fpsyg.2019.02843

**Published:** 2019-12-13

**Authors:** Andrés Fuster-Guilló, María Luisa Pertegal-Felices, Antonio Jimeno-Morenilla, Jorge Azorín-López, María Luisa Rico-Soliveres, Felipe Restrepo-Calle

**Affiliations:** ^1^Department of Computer Science and Technology, University of Alicante, Alicante, Spain; ^2^Department of Development Psychology and Teaching, University of Alicante, Alicante, Spain; ^3^Department of Systems and Industrial Engineering, Universidad Nacional de Colombia, Bogotá, Colombia

**Keywords:** gamification, serious games, motivation, teaching-learning, computer engineering

## Abstract

Gamification methods adapt the mechanics of games to educational environments for the improvement of the teaching-learning process. Serious games play an important role as tools for gamification, in particular in the context of software engineering courses because of the idiosyncratic nature of the topic. However, the studies on the improvement of student performance resulting from the use of gamification and serious games in courses with different contexts are not conclusive. More empirical research is thus needed to obtain reliable results on the effectiveness, benefits and drawbacks. The overall objective of this work is to study the benefits generated by serious games in the teaching-learning process of Computer Engineering degrees, analyzing the impact on the motivation and student satisfaction, as well as on the learning outcomes and results finally achieved. To this end, an intervention is proposed in the subject of Computer Architecture based on two components covering theoretical and practical sessions. In the theoretical sessions, a serious game experience using Kahoot has been introduced, complementing the master classes and class exercises. For the practical sessions, the development of projects with groups of students has been proposed, whose results in terms of computer performance can be compared through a competition (hackathon). Evaluation of the serious game-based intervention has been approached in terms of student satisfaction and motivation, as well as improved academic performance. In order to assess student satisfaction, surveys have been used to assess the effect on student motivation and satisfaction. For the evaluation of academic performance, a comparative analysis between an experimental and a control group has been carried out, noting a slight increase in the experimental group students’ marks.

## Introduction

The present work aims to contribute to the study of the potential benefits of serious games on the teaching-learning process in a higher education context, and specifically in relation to computer engineering studies. A dual experience is proposed which considers the particularities of the theoretical and practical components. The impact on student motivation and satisfaction of a serious game-based learning experience using Kahoot is analyzed, as well as on the learning outcomes finally achieved.

Gamification has become more relevant at the end of the first decade of the 21st century. Some authors define it as the use of typical elements of games in contexts outside the game environment (Deterding et al., 2011). According to Villagrasa et al. (2014) the main objective of gamification is to increase commitment and motivation. Gamification has been widely and successfully used in marketing to influence consumer behavior (Zichermann and Cunningham, 2011). In education, gamification techniques transfer the mechanics of games to educational environments with the aim of improving motivation, and consequently the teaching-learning process (Lee and Hammer, 2011; De-Marcos et al., 2017). The aim is to encourage interaction between teacher and student in order to increase motivation, leading to an improvement in the capacity to assimilate knowledge and acquire skills.

Experiences based on gamification have had an impact on basic and intermediate levels of education and are gradually being incorporated into university environments (Wiggins, 2016) as a response to a demand to understand the learning processes of younger generations. The work of Subhash and Cudney (2018) conducts an extensive review of gamification in the higher education environment. Moreover, the use technology in education may be a way of improving overall performance of Higher Education Institutions (HEI), due to possibly also improving professors’ satisfaction and motivation (Lytras et al., 2019); and may have an impact on the overall broad question of HEI sustainability (Visvizi and Daniela, 2019).

The work by Caton and Greenhill (2014) shows how awards motivate students to produce higher quality results and attempt challenging tasks. The methods most commonly used in gamification in the context of higher education are those based on points, badges, leader boards, levels, missions or challenges. The work of Mayer et al. (2014) explores the contributions and weaknesses of Game Based Learning (GBL) and Serious Games (SGs). Serious games play an important role as components for gamification in learning process. Tools to perform interactive quizzes like kahoot have proven their effectiveness on student motivation (Orhan Göksün and Gürsoy, 2019). However, studies on the improvement of student performance resulting from the use of gamification and serious games in different subject contexts are not conclusive (Subhash and Cudney, 2018).

The work of Alhammad and Moreno (2018) provides a systematic mapping of the state of the art in gamification in software engineering studies. This study introduces a number of interesting conclusions highlighting the greater importance of gamification in the educational process of software engineering due to the idiosyncrasy of the subject area. This study concludes that more empirical research is needed to arrive at reliable results on the effectiveness, benefits and drawbacks of gamification. It is worth remembering Gartner’s warning that around 80% of gamification applications will not meet business objectives, mainly because the processes have been inappropriately adapted to gamification. Moreover, the works performed by Petri et al. (2018) and Marín et al. (2019) highlight the relevance of serious games especially in this area.

From the review of the state of the art we can infer an interest to contribute with empirical studies to the clarification of the benefits of gamification and serious games, especially in the context of the higher level studies of computer engineering where the idiosyncrasies of the subject area create a case of special application interest.

For these reasons, an intervention in the subject of Computer Architecture within the Computer Engineering Degree at the University of Alicante is proposed as an objective. It is further proposed to use a double method to consider the specificities of the theoretical and practical sessions. In the case of the theoretical sessions, a serious game with interactive questionnaires is proposed by using “Kahoot” (Licorish et al., 2018), an online free game-based learning platform which allows the creation of different questions types like multiple choice quizzes, discussion questions, or surveys. The gamification method of the practical sessions is based on competitions in “hackathon” format, specifically the “CUDATHON” competition. Once the intervention has been developed, the benefits of the serious game-based experience in terms of student motivation and satisfaction, as well as in terms of learning outcomes, can be analyzed.

The rest of the paper is structured as follows: in the second section “Materials and Methods” the context is explained and the participants of the intervention, the instruments of the proposal and the procedure are also detailed. In the third section “Results,” the data obtained in the intervention are reported. Finally, in the fourth section “Discussion and Conclusion” the data obtained are analyzed and the conclusions reached are summarized.

## Materials and Methods

Once the state of the art has been reviewed and the general and specific objectives of the research defined, this section explains the methodology of case of study (Wohlin et al., 2003) that uses the satisfaction survey as a tool to obtain student feedback. It describes, in detail, the teaching context and objects of study, the instrument to be used and the procedures planned for its development, including the proposed serious game tool.

### Description of the Context and Participants

The subject in which the serious game experience is developed is “Computer Architecture” from the “Degree in Computer Engineering” at the University of Alicante. This course is compulsory in the second semester of the second year. It has six ECTS credits (1.2 theoretical, 1.2 practical, and 3.6 non-presential load). This means 30 classroom hours of theory and 30 classroom hours of practice, organized into 15 sessions of 2 h for both theoretical and practical elements.

The 15 2-h theoretical sessions aim to provide the student with knowledge on computer performance assessment, computer concepts and models, instruction set design, instruction level parallelism, segmentation, memory, and I/O performance. To this end, content is organized into six topics. The teaching methodology combines traditional resources such as master classes with highly experimental mechanisms and student participation. In this context, in the academic year 2018–2019 games have been introduced into the theoretical sessions using “gaming” tools (Kahoot) to encourage student participation and motivation. The theoretical part is evaluated by means of tests during the course and a final examination of problems. In the academic year 2018–2019, gaming leader boards have been incorporated into the theoretical evaluation with the possibility of students obtaining up to one extra point for participating in the games.

The 15 2-h practical sessions aim to provide the student with the skills to be able to implement test programs to evaluate specific aspects of the computer; to use standard benchmarks to make performance reports; to design optimal software solutions taking advantage of the parallelism provided by the architectures (80 × 86, SIMD, MMX, SSE, CUDA). For the development of the practices, a project organized in three phases is proposed. Phase I deals with performance evaluation. Phase II is about taking advantage of the parallelism of the architectures through the use of 80 × 86, SIMD, MMX, SSE technologies. Phase III deals with the use of massively parallel architectures with technologies such as CUDA. Individual work is carried out to guarantee the individual acquisition of skills, along with group exercises to develop organization and integration capabilities within a work group. In this context, since the academic year 2015–2016 a “hackathon” competition has been introduced to encourage student participation and motivation. Different groups of students compete with the solution then implemented using CUDA technology which achieves a better performance in solving a given problem. The competition is organized in a day called “CUDATHON.” The individual part of the student’s work is evaluated by means of multiple-choice tests. The group part is evaluated by means of reports and classroom exhibitions. Since 2015–2016 the “CUDATHON” has been incorporated into the practical evaluation with the possibility of obtaining an extra point.

The number of students enrolled in Computer Architecture is usually around 140 organized into four theory groups and seven practice groups. The theory groups are taught in different languages: two in Spanish (morning and afternoon), one in Valencian and one in English (HAP: High Academic Performance). Of the seven practice groups there are five in Spanish (three in the morning and two in the afternoon), one in Valencian and one in English (HAP: High Academic Performance).

### Instruments

At this point, it is appropriate to recall the specific measurement objectives of the intervention. On the one hand, it is intended to measure the effect of Kahoot-based intervention on motivation and academic performance; on the other hand, to measure the effect of CUDATHON-based experience. These last results are not detailed in the present work, given that the CUDATHON experience began the 2015–2016 academic year, requiring comparison with data from previous courses in which practices with assimilable CUDA were not developed. For this reason, in this paper we focus on detailing the effects of Kahoot-based experience with the following objectives:

(1) To measure the effect of Kahoot-based intervention on student motivation through satisfaction surveys – To cover this objective, a satisfaction survey has been prepared using the Kahoot platform itself. This survey has been carried out in the tenth theoretical session, with nine Kahoots carried out, which allows the student to formulate their own opinion on the interest and details of the use of Kahoot in the classroom. Specifically, the satisfaction survey asks seven questions with the following answers that appear in Table 1.
(2) To measure the effect of Kahoot-based intervention on learning outcomes by analysing the results of theoretical evaluation: To measure the effect on academic outcomes it is proposed to compare the results of the theoretical evaluation of the present academic year 2018–2019 with the results of the previous academic year 2017–2018 in which the experience with Kahoot was not developed. Therefore, the proposal contemplates the comparison of the marks of this test with respect to the previous course.

**TABLE 1:** Satisfaction survey questions and answers about Kahoot use.

**Q/Resp**	**Question**	**Response**
Q1Resp1	When I play Kahoot	“I have fun but I don’t learn”
Q1Resp2		“I have fun and I learn”
Q1Resp3		“I don’t have fun but I learn”
Q1Resp4		“I don’t have fun or learn”
Q2Resp1	Making Kahoots helps me reinforce what I learned in class	“It doesn’t help me at all”
Q2Resp2		“It helps me a little”
Q2Resp3		“It helps me”
Q2Resp4		“It helps me a lot”
Q3Resp1	Making Kahoots motivates me to learn the subject	“Nothing”
Q3Resp2		“Little”
Q3Resp3		“Quite a lot”
Q3Resp4		“A lot”
Q4Resp1	I prefer to do the Kahoot	“As soon as class starts”
Q4Resp2		“In the middle of class”
Q4Resp3		“At the end of class”
Q5Resp1	I would like the Kahoot’s length to be	“Short (<5 min)”
Q5Resp2		“Medium (between 5 and 15 min)”
Q5Resp3		“Long (>15 min)”
Q6Resp1	I prefer the teacher to use to explain the theory	“Exclusively his explanation”
Q6Resp2		“His explanation combined with Kahoot”
Q6Resp3		“His explanation combined with practical exercises”
Q6Resp4		“His explanation combined with Kahoot and practical exercises”
Q7Resp1	In general, I consider Kahoot to be	“Unnecessary”
Q7Resp2		“Unimportant”
Q7Resp3		“Necessary”
Q7Resp4		“Essential”

The statistic Alfa de Cronbach has been chosen to carry out the reliability analysis of the survey. According to the recommendations of Gliem and Gliem (2003), Cronbach alpha coefficients can be evaluated as follows: alpha > 0.9 excellent; alpha > 0.8 good; alpha > 0.7 acceptable; alpha > 0.6 questionable; alpha > 0.5 poor; and alpha < 0.5 unacceptable. On the other hand, Nunnally (1978) mentions that in a standard exploratory analysis, an estimated alpha coefficient of 0.7 is considered adequate. The reliability result, after applying Cronbach’s Alfa statistic with the instrument items was 0.75 which is acceptable considering the low number of items and the previous considerations.

Regarding the validation of the instrument, it was carried out by means of the Content Validity Index (CVI) proposed by Lawshe (1975). Under this validity index, the variables or items of an instrument are subject to expert review and are quantified as follows:

$$\text{CVI}=\frac{\text{ne}-N/2}{N/2}$$

ne: number of experts who rate the item favourably; *N*: total number of experts valuing the item.

In this research, nine professors from outside the research validated the instrument. In this sense, eight experts rated all the items favourably and one expert rated all the items positively except for Q5 item. In order for the instrument to be validated with nine experts, a CVI of 0.75 or higher is required for all of its items and in this case the CVI was 0.78 for all the items. Therefore, the instrument was validated under this index.

### Procedure

As mentioned, in the academic year 2018–2019 the incorporation of games into the theoretical sessions was formalized using serious games tools such as Kahoot. The aim is to complement the masterclass method by incorporating quizzes to encourage student participation and motivation. In previous courses this type of activity was already carried out but in an unplanned way, nor generalized in relation to the subject area. In the present 2019 academic year the systematized realization of “quizzes” has been planned in all the theoretical sessions of all the groups of the subject. The Kahoot tool is an online platform that allows the development by the teacher of questionnaire “quizzes” that can be raised interactively during class sessions, to get feedback from students on the assimilation of some concept previously exposed. The Kahoot platform enables students to answer a series of questions with answers in the form of options, so that during the game everyone can observe for each question the number of student answers to each of the options, as well as the ranking achieved by the participants according to the points obtained by correct answers.

Through the use of the Kahoot tool, an interactive questionnaire linked to each of the 15 theoretical sessions has been proposed. These questionnaires are designed to be developed in the last 15 min of each theoretical session. The questions are short statements (about 20 words) and four answer options. Depending on the topic explained in class, the questions may or may not require calculations, so response times can range from 10 to 90 s. The number of questions ranges from 5 to 12 depending on the length of the response times.

A total of 15 Kahoots (with their corresponding translations into Valencian and English) have been prepared for use in each of the 15 theoretical sessions. Table 2 shows the titles of the 15 Kahoots proposed and their links to thematic units:

**TABLE 2:** List of Kahoot quizzes for each theoretical session.

**Kahoot/Session**	**Title**	**Lesson**
Kahoot1: Session1	Initial concepts	T1. Introduction
Kahoot2: Session2	Performance	T2. Performance
Kahoot3: Session3	Amdahl	T2. Performance
Kahoot4: Session4	CPU Performance	T2. Performance
Kahoot5: Session5	Instruction Set Architecture ISA	T3. Instruction Set Architecture
Kahoot6: Session6	Instruction Set Architecture ISA 2	T3. Instruction Set Architecture
Kahoot7: Session7	Introducing segmentation	T4. Segmentation
Kahoot8: Session8	Segmented performance	T4. Segmentation
Kahoot9: Session9	Pipeline segmentation	T4. Segmentation
Kahoot10: Session10	Satisfaction survey	
Kahoot11: Session11	Pipeline segmentation 2	T4. Segmentation
Kahoot12: Session12	Pipeline segmentation 3	T4. Segmentation
Kahoot13: Session13	Memory 1	T5. Memory
Kahoot14: Session14	Memory 2	T5. Memory
Kahoot15: Session15	Input Output	T6. Input Output

The theoretical part represents 50% of the overall mark for the subject. This part is assessed by means of two multiple-choice tests (30% of the theoretical assessment) with theoretical questions test1 (topics 1 and 2) and test2 (topics 3, 4, and 5) and a final exam of problems (70% of the theoretical assessment). In the academic year 2018–2019, gaming leader boards have been incorporated into the theoretical evaluation through the possibility of obtaining one extra point for participating in the games. In each Kahoot of the theoretical sessions the students accumulate kahoot-points for the leader board, depending on the number of correct answers. The one who accumulates the most kahoot-points, once all the kahoots have been completed, gets the extra point. The rest of the students obtain a fraction of the extra point calculated according to their kahoot-points in relation to the kahoot-points for the first classified. The leader board of accumulated kahoot-points is shown in each session.

## Results

The following section presents the results of the research. First, the effect of Kahoot-based experience on student motivation is analyzed through their responses to the satisfaction survey. This survey was carried out during the 10th theoretical session and was answered by a total of 65 students.

**FIGURE 1:**
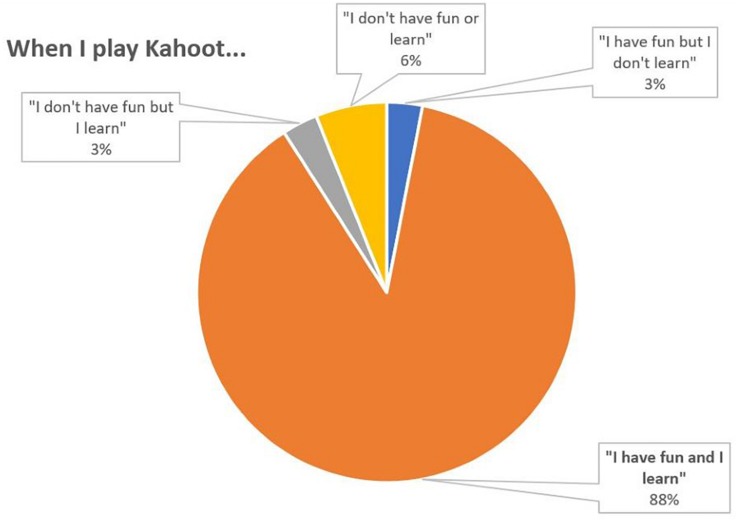
Percentage distribution of responses to the question 1.

**FIGURE 2:**
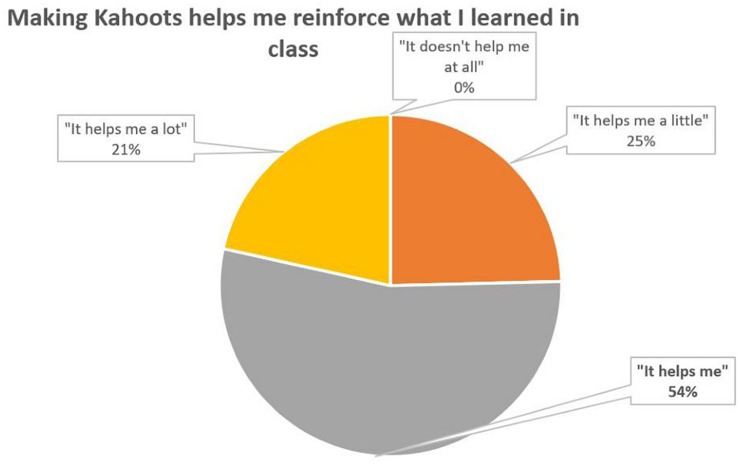
Percentage distribution of responses to the question 2.

It is observed that most students (88%) “have fun and learn” when they play Kahoot (Figure 1), which is a clear positive indicator of motivation. In addition, does making Kahoots reinforce what you have learned in class? Most of them (54%) (Figure 2) answer that it helps them.

**FIGURE 3:**
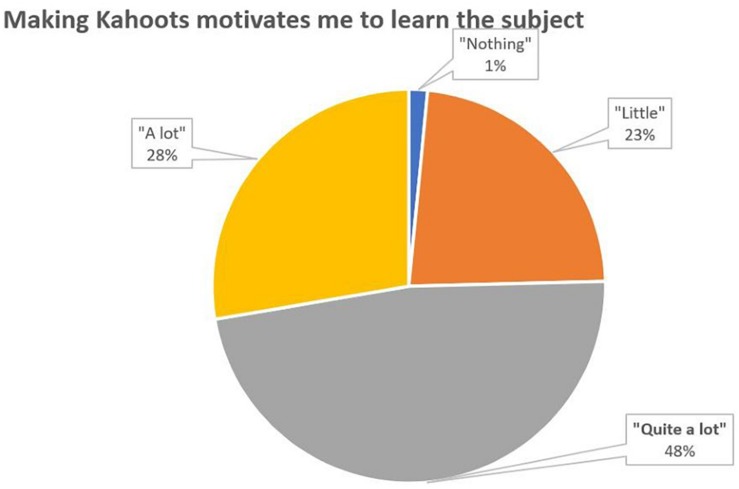
Percentage distribution of responses to the question 3.

To the direct question about motivation, does Kahoots motivate me to learn the subject? The majority answer is “Quite a lot” (48%) (Figure 3).

**FIGURE 4:**
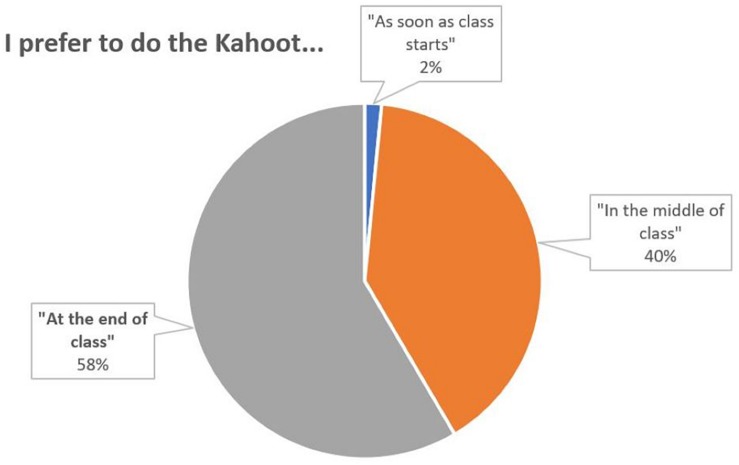
Percentage distribution of responses to the question 4.

**FIGURE 5:**
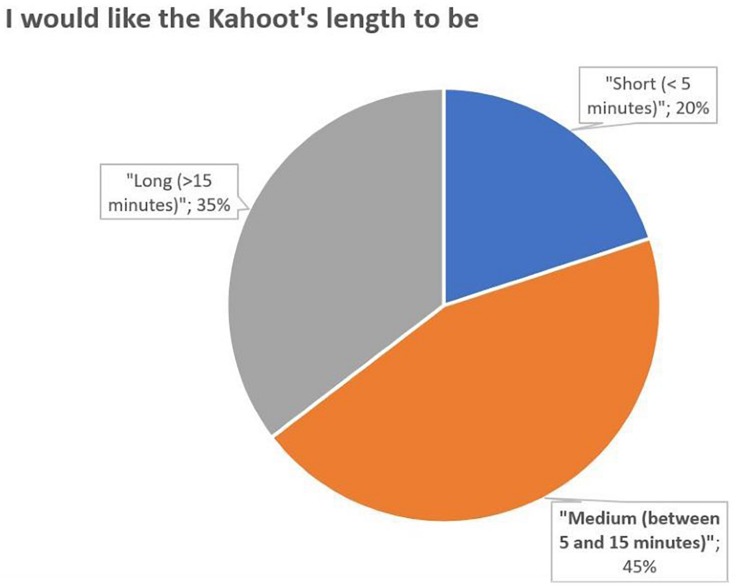
Percentage distribution of responses to the question 5.

Questions 4 and 5 refer to the dynamics of the questionnaires, asking for the best time to perform them Q4 and the preferred duration Q5. It is observed that the students prefer to ask the questionnaires at the end of each session (58%) (Figure 4). It can also be seen that the length preferred by the students for the questionnaires is between 5 and 15 min (Figure 5).

**FIGURE 6:**
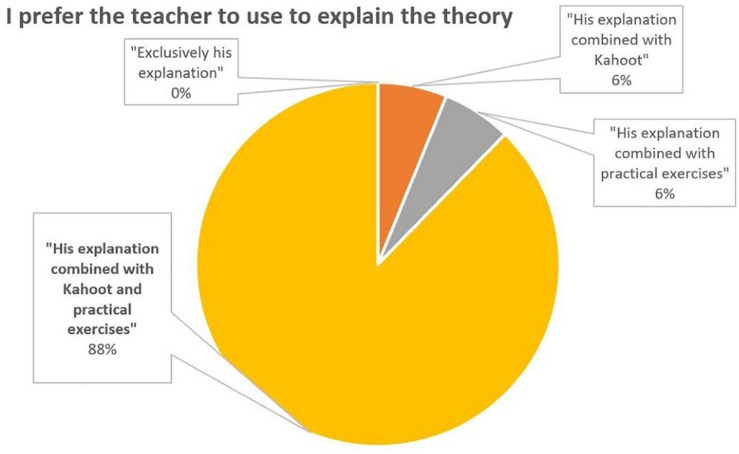
Percentage distribution of responses to the question 6.

The students also show a clear preference for a teaching methodology that makes use of theoretical explanations combined with Kahoots and practical exercises (88%) (Figure 6).

**FIGURE 7:**
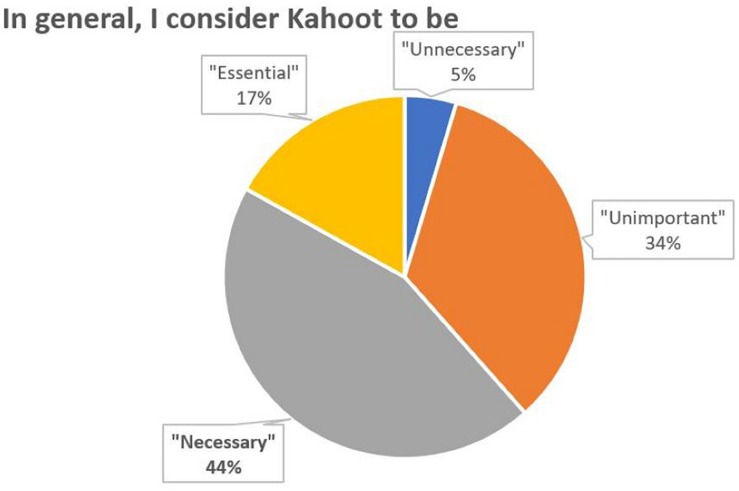
Percentage distribution of responses to the question 7.

Regarding the generic assessment question on the use of Kahoot, it should be noted that 61% of students consider the use of Kahoot to be essential or necessary (Figure 7).

Finally, in order to measure the effect of Kahoot-based experience on learning outcomes, average results are provided by theory group for the current academic year 2018–2019 (called experimental group) in relation to the results for the previous academic year 2017–2018, in which the serious game experience was not developed (called control group). In both experimental and control groups, the assignment of tasks and the assessment methods were the same.

To assess the program’s impact on student performance, two grades obtained by both groups were compared: prior to (pretest) and after the experiment (posttest). The pretest consisted of the assessment of a preliminary examination made in the very beginning of the course (first week) to test the initial knowledge of the students for facing the subject. The posttest consisted of the final examinations of the course (after week 15). Belonging to one group or the other was the independent factor or variable, and the scores obtained by the students in these examinations were the criteria or dependent variables.

The statistical procedure used the general linear model with repeated measures, with the score obtained for the examinations being taken as the dependent variable. The time of assessment (pretest and posttest) was used as the intra-subject factor; and participation in the experiment (belonging to the experimental or control group) was the inter-subject factor. All statistical analyses were conducted using SPSS (version 24.0).

The values of the inter-subject test (see Table 3) indicate that the means of all observations differ from 0 because the tests have been shown to be significant (*p* < 0.000) for intersection but not for group belonging (*p* = 0.244). This finding confirms that there are no initial significant differences between the two groups of students.

**TABLE 3:** Test of inter-subject effects.

**Source**	**Type III error**	**Gl**	***F***	**Sig.**
Intersection	12888.686	1	1849.842	0.000
Group	9.516	1	1.366	0.244
Error	1079.955	155		

For the implementation of the program, Table 4 shows the test for intra-subject effects. The values resulting from the test show that the effect of the interaction between the time of assessment (pretest and posttest) and the intervention is significant (*p* = 0.000). The observed power is 0.989, rejecting the null hypothesis of equality of means. The effect size (η^2^), proportion of total variability attributable to a factor (Gardner, 2003), or the magnitude of the difference between one time and another (Ledesma et al., 2008), resulting from the interaction between the time of the assessment and the implementation of the program is 0.105.

**TABLE 4:** Test of intra-subject effects.

**Source**	**Type III error**	**Gl**	***F***	**Sig.**	**η^2^ partial**	**Ob. Power**
Apl	26.390	1	11.572	0.001	0.069	0.922
Gr × Apl	41.450	1	18.176	0.000	0.105	0.989
Error	353.469	155				

Finally, to test whether there is any difference between the experimental group and control group, at the time of pretest and posttest, a Student’s *t*-test on the difference in means was conducted, Table 5, which shows that there were no significant differences at the time of pretest (*p* = 0.343). This finding could mean that both groups began in comparable situations, which was already suggested by the inter-subject test. For the posttest, the test shows a significant difference between the two groups (*p* = 0.000); this difference is 1.08 out of 10 points higher in the experimental group.

**TABLE 5:** Student’s *t*-test on the difference of means between the experimental and control groups.

**Moment**	***t***	**Gl**	**Sig.**	**Diff.^∗^**	**Std. dev.**
PRE	0.951	155	0.343	–0.380	0.399
POST	–4.323	155	0.000	1.079	0.249

*^∗^Difference expressed as experimental group scores minus control group scores.*

**FIGURE 8:**
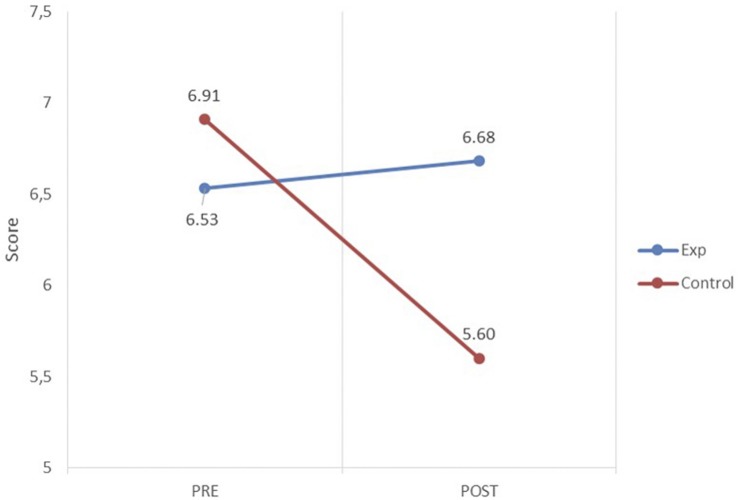
Academic performance score (out of a maximum of 10) of the groups at pretest (PRE) and posttest (POST).

Figure 8 shows the scores obtained by both groups before and after the intervention. In the posttest, the experimental group, who had used the gaming strategy, had higher scores, whereas the control group who had had no gaming interaction had worse performance.

## Discussion and Conclusion

The purpose of this case study was to evaluate a game-based intervention designed to improve student motivation and satisfaction, as well as the results and outcomes of learning and the evaluation of student satisfaction after the intervention (Wieringa, 2010).

According to all measures the introduction of Kahoot in theory classes appears to have been succeeded. Most students reported a positive perception and a positive attitude toward learning (56% believe that it reinforces what have been learned and 48% believe that it motivates a lot to learn, Figures 2, 3). In general, students felt that the use of Kahoot is essential or necessary (61% according to Figure 7). Students overwhelmingly showed preference in sharing theoretical lesson with Kahoot and practical exercises (88% according to Figure 6). In addition, the improved academic performance in the experimental group into which Kahoot was introduced further supports the success of the intervention.

This work proposes a game-based experience applied in the higher educational environment of Computer Engineering based on a double method that contemplates the particularities of the theoretical and practical components of the subject. The aim of the study is to measure the improvement in student motivation and academic performance as a result of introducing the game-based experience.

In the theoretical sessions a serious game-based experience has been introduced. Specifically, interactive quizzes using the Kahoot tool have been used, incorporating questionnaires for each of the theoretical sessions. In addition, leader boards have been used to encourage participation. The result allows us to state that the serious game experience is clearly positive from the point of view of the motivation and degree of satisfaction of the student, which is clearly observed in the satisfaction survey carried out on them. This conclusion is consistent with most of the gamification and game-based experiences reviewed in the state of the art, in which a clear impact on motivation is observed (Villagrasa et al., 2014).

The gamification experience of the practical sessions is based on the proposal of competitive practices, in which groups of students compete to find the best solution to some of the problems proposed, interacting with other groups that solve the same problem. This competition called CUDATHON culminates with a hackathon format. Nevertheless, the analysis of the data provided in the results section have not been influenced by CUDATHON, since this experience has been developed in the same way in the two academic courses that are compared in the study. Although our perception in the classroom allows us to qualitatively affirm the positive acceptance of CUDATHON by students, we cannot infer or quantify its effect on motivation or academic performance.

Regarding the impact of the serious game experience on learning outcomes, the experimental results show different findings for the experimental group and the control group. First, when focusing exclusively on traditional teaching (control group, and experimental group before the pretest), a worse group performance can be observed. This decrease is significant in comparison with the experimental group, as shown in the intra-subject test in Table 4. This decrease may be due to the fact that the posttest is more difficult than the pretest, which is conducted at a time when student knowledge is still limited. For the experimental group, the usual tendency of obtaining lower scores than the initial test is not seen. Despite the difficulty of the posttest, there was a mild improvement, and the relatively improved results achieved by the experimental group are considered to be relevant.

Studies on the improvement of academic performance as a consequence of the use of gamification and serious games differ depending on the application context (Subhash and Cudney, 2018). The main contribution of this paper is to reinforce the idea of the improvement of learning outcomes as a consequence of introducing a serious game experience in the context of computer engineering courses at the higher education level.

Because Kahoot is widely used, the experience can be easily extrapolated to other educational fields. The benefits in other areas such as social sciences or humanities can be comparable to those obtained by this research. In this way it is proposed as future work to replicate this experience in other degrees and make a comparison with the results obtained in this research.

## Data Availability Statement

The datasets generated for this study are available on request to the corresponding author.

## Ethics Statement

The studies involving human participants were reviewed and approved by the University of Alicante. The patients/participants provided their written informed consent to participate in this study.

## Author Contributions

AF-G, MP-F, and AJ-M: conceptualization, methodology, and reviewing and approving the final manuscript. JA-L, FR-C, and MR-S: experimentation and validation. AF-G and MR-S: writing and reviewing.

## Conflict of Interest

The authors declare that the research was conducted in the absence of any commercial or financial relationships that could be construed as a potential conflict of interest.
